# Author Correction: Effects of electroconvulsive therapy on hippocampal longitudinal axis and its association with cognitive side effects

**DOI:** 10.1038/s43856-026-01390-3

**Published:** 2026-01-23

**Authors:** Olga Therese Ousdal, Miklos Argyelan, Maarten Laroy, Amit Anand, Filip Bouckaert, Joan A. Camprodon, Marta Cano, Narcis Cardoner, Udo Dannlowski, Annemiek Dols, Louise Emsell, Randall Espinoza, Kaat Hebbrecht, René Hurlemann, Martin Jorgensen, Maximilian Kiebs, Taishiro Kishimoto, Katherine L. Narr, Pia Nordanskog, Nils Opel, Ronny Redlich, Didi Rhebergen, Alexander Sartorius, Didier Schrijvers, Pascal Sienaert, Carles Soriano-Mas, Akihiro Takamiya, Freek ten Doesschate, Indira Tendolkar, Mikel Urretavizcaya, Linda van Diermen, Philip van Eijndhoven, Guido van Wingen, Jeroen van Waarde, Mathieu Vandenbulcke, Joey Verdijk, Benjamin S. C. Wade, Yrondi Antoine, Njål Brekke, Joan Prudic, Shawn McClintock, Ute Kessler, Hauke Bartsch, Ketil Odegaard, Jan Haavik, Åsa Hammar, Christopher Abbott, Leif Oltedal

**Affiliations:** 1 Department of Biomedicine, The Faculty of Medicine, University of Bergen, Bergen, Norway; 2 Mohn Medical Imaging and Visualization Center, Department of Radiology, Haukeland University Hospital, Bergen, Norway; 3 Feinstein Institutes for Medical Research, Institute of Behavioral Science, Manhasset, NY USA; 4 The Zucker Hillside Hospital, Glen Oaks, NY USA; 5 Department of Neurosciences and Neuropsychiatry, KU Leuven, Leuven Brain Institute, Leuven, Belgium; 6 Harvard Medical School, Boston, MA USA; 7 Brigham and Women’s Hospital, Neuroscience Center, Boston, MA USA; 8 Geriatric Psychiatry, University Psychiatric Center KU Leuven, Leuven, Belgium; 9 Division of Neuropsychiatry and Neuromodulation, Department of Psychiatry, Massachusetts General Hospital, Harvard Medical School, Boston, MA USA; 10 Sant Pau Mental Health Research Group, Institut d’Investigació Biomédica Sant Pau, Hospital de la Santa Creu I Sant Pau, Barcelona, Spain; 11 Department of Psychiatry and Forensic Medicine, Universitata Autònoma de Barcelona, Barcelona, Spain; 12 CIBERSAM, Carlos III Health Institute, Madrid, Spain; 13 Institute for Translational Psychiatry, University of Muenster, Muenster, Germany; 14 Amsterdam UMC location Vrije Universiteit Amsterdam, Psychiatry, Neuroscience, Amsterdam, The Netherlands; 15 Department of Psychiatry, UMC Utrecht Brain Center, University Medical Center Utrecht, Utrecht, The Netherlands; 16 Departments of Neurology and Psychiatry and Biobehavioral Sciences, University of California Los Angeles, Los Angeles, CA USA; 17 Department of Psychiatry, University Psychiatric Center KU Leuven, Leuven, Belgium; 18 Academic Center for ECT and Neuromodulation (AcCENT) University Psychiatric Center, KU Leuven, Kortenberg, Belgium; 19 Department of Psychiatry and Psychotherapy, School of Medicine & Health Sciences, Carl von Ossietzky University of Oldenburg, Oldenburg, Germany; 20 Psychiatric Center Copenhagen and Department of Clinical Medicine, University of Copenhagen, Copenhagen, Denmark; 21 Department of Psychiatry and Psychotherapy, University Hospital Bonn, Bonn, Germany; 22 Hills Joint Research Laboratory for Future Preventive Medicine and Wellness, Keio University School of Medicine, Tokyo, Japan; 23 Center for Social and Affective Neuroscience (CSAN), Department of Biomedical and Clinical Sciences, Linköping University, Linköping, Sweden; 24 Department of Psychiatry in Linköping, Linköping, Sweden; 25 Department of Psychiatry, University of Jena, Jena, Germany; 26 Institute of Translational Psychiatry, University of Muenster, Muenster, Germany; 27 Department of Psychology, University of Halle, Halle, Germany; 28 German Center for Mental Health (DZPG), Halle-Jena-Magdeburg, Magdeburg, Germany; 29 Amsterdam Public Health Research Institute, Department of Mental Health, Amsterdam UMC, Amsterdam, The Netherlands; 30 Department of Research, GGZ Central Innova, Amersfoort, The Netherlands; 31 Department of Psychiatry and Psychotherapy, Central Institute of Mental Health (CIMH), Medical Faculty Mannheim, University of Heidelberg, Heidelberg, Germany; 32 Collaborative Antwerp Psychiatric Research Institute, University of Antwerp, Antwerpen, Belgium; 33 University Psychiatric Hospital Duffel, Duffel, Belgium; 34 Department of Psychiatry, Bellvitge University Hospital, Bellvitge Biomedical Research Institute- IDIBELL, Barcelona, Spain; 35 Department of Social Psychology and Quantitative Psychology, Universitat de Barcelona, Barcelona, Spain; 36 Department of Psychiatry, Rijnstate Hospital Arnhem, Arnhem, The Netherlands; 37 Donders Institute for Brain, Cognition and Behavior, Department of Psychiatry, Radboud University Nijmegen, Nijmegen, The Netherlands; 38 Department of Clinical Sciences, Bellvitge Campus, Universitat de Barcelona, Barcelona, Spain; 39 Psychiatric Hospital Bethanië, Zoersel, Belgium; 40 Department of Psychiatry, Amsterdam UMC Location University of Amsterdam, Amsterdam, The Netherlands; 41 Amsterdam Neuroscience, Amsterdam, The Netherlands; 42 Service de Psychiatrie et Psychologie Médicale, Centre Expert Dépression Résistante, Foundation Fondamental, CHU, Toulouse, France; 43 Toulouse Neuroimaging Center, Université de Toulouse, INSERM, Toulouse, France; 44 Department of Psychiatry, Colombia School of Medicine, New York, NY USA; 45 Division of Psychology, Department of Psychiatry, UT Southwestern Medical Center, Dallas, TX USA; 46 Department of Clinical Medicine, University of Bergen, Bergen, Norway; 47 Division of Psychiatry, Haukeland University Hospital, Bergen, Norway; 48 Department of Computer Science, University of Bergen, Bergen, Norway; 49 Department of Biological and Medical Psychology, University of Bergen, Bergen, Norway; 50 Department of Clinical Sciences Lund Psychiatry, Faculty of Medicine, Lund University, Lund, Sweden; 51 Department of Psychiatry, Skåne University Hospital, Lund, Sweden; 52 Department of Psychiatry, University of New Mexico, Albuquerque, NM USA

**Keywords:** Adverse effects, Depression

Correction to: *Communications Medicine* 10.1038/s43856-025-01120-1, published online 01 October 2025

In the version of the article initially published, the first name of Maximilian Kiebs was misspelled (Maximillian), and is now amended in the HTML and PDF versions of the article.

