# Supplementary material for: Effects of electroconvulsive therapy on hippocampal longitudinal axis and its association with cognitive side effects
Source: Commun Med (Lond). 2025 Oct 1;5:409. doi: 10.1038/s43856-025-01120-1 (PMC12489027; doi:10.1038/s43856-025-01120-1)
Supplement: Supplementary file 2 — Supplementary Information [file 43856_2025_1120_MOESM2_ESM.pdf]

Effects of Electroconvulsive Therapy on hippocampal longitudinal axis and its association with cognitive side effects

Olga Therese Ousdal[1,2], Miklos Argyelan[3,4], Maarten Laroy[5], Amit Anand [6,7], Filip Bouckaert[8], Joan A. Camprodon[9], Marta Cano [9], Narcis Cardoner [10,11,12], Udo Dannlowski[13], Annemiek Dols[14,15], Louise Emsell [5], Randall Espinoza[16], Kaat Hebbrecht[17,18], René Hurlemann[19], Martin Jorgensen[20] , Maximillian Kiebs[19, 21], Taishiro Kishimoto[22], Katherine L. Narr[16] , Pia Nordanskog[23,24] , Nils Opel[25,26], Ronny Redlich[26,27,28], Didi Rhebergen[29,30], Alexander Sartorius[31], Didier Schrijvers[32,33], Pascal Sienaert[18] , Carles Soriano-Mas[34,35,36], Akihiro Takamiya[22], Freek ten Doesschate[37], Indira Tendolkar[38], Mikel Urretavizcaya[36,39,40], Linda van Diermen[41,42], Philip van Eijndhoven[38], Guido van Wingen[43,44] , Jeroen van Waarde[37], Mathieu Vandenbulcke[5,8] , Joey Verdijk[37], Benjamin S.C. Wade[9] , Yrondi Antoine[45,46], Njål Brekke[2] , Joan Prudic[47], Shawn McClintock[48], Ute Kessler[49,50] , Hauke Bartsch[2,51], Ketil Odegaard[49,50], Jan Haavik[1,50], Åsa Hammar[52,53,54] , Christopher Abbott[55], Leif Oltedal[2,49]

[1]Department of Biomedicine, The Faculty of Medicine, University of Bergen, Bergen, Norway
[2] Mohn Medical Imaging and Visualization Center, Department of Radiology, Haukeland University Hospital, Bergen, Norway
[3]Feinstein Institutes for Medical Research, Institute of Behavioral Science, Manhasset, New York, USA
[4]The Zucker Hillside Hospital, Glen Oaks, New York, USA
[5]KU Leuven, Leuven Brain Institute, Department of Neurosciences, Neuropsychiatry, Leuven, Belgium
[6] Harvard Medical School, Boston, USA
[7]Brigham and Women's hospital, Neuroscience Center, Boston, USA
[8] Geriatric Psychiatry, University Psychiatric Center KU Leuven, Leuven, Belgium
[9]Division of Neuropsychiatry and Neuromodulation, Department of Psychiatry, Massachusetts General Hospital, Harvard Medical School. Boston, USA.
[10]Sant Pau Mental Health Research Group, Institut d'Investigació Biomédica Sant Pau, Hospital de la Santa Creu I Sant Pau, Barcelona, Spain
[11]Department of Psychiatry and Forensic Medicine, Universitata Autònoma de Barcelona, Barcelona, Spain
[12]CIBERSAM, Carlos III Health Institute, Madrid, Spain
[13]Institute for Translational Psychiatry, University of Muenster, Muenster, Germany
[14]Amsterdam UMC location Vrije Universiteit Amsterdam, Psychiatry, Neuroscience, Amsterdam, The Netherlands
[15] Department of Psychiatry, UMC Utrecht Brain Center, University Medical Center Utrecht, Utrecht, The Netherlands
[16]Departments of Neurology and Psychiatry and Biobehavioral Sciences, University of California, Los Angeles, Los Angeles, California, USA
[17]University Psychiatric Center KU Leuven, Department of Psychiatry, Leuven, Belgium
[18]Academic enter for ECT and Neuromodulation (AcCENT) University Psychiatric Center, KU Leuven, Kortenberg, Belgium
[19]Department of Psychiatry and Psychotherapy, School of Medicine & Health Sciences, Carl von Ossietzky University of Oldenburg, Oldenburg, Germany
[20] Psychiatric Center Copenhagen and Department of Clinical Medicine, University of Copenhagen, Copenhagen, Denmark
[21]Department of Psychiatry and Psychotherapy, University Hospital Bonn, Bonn, Germany
[22]Hills Joint Research Laboratory for Future Preventive Medicine and Wellness, Keio University School of Medicine, Tokyo, Japan
[23]Center for Social and Affective Neuroscience (CSAN), Department of Biomedical and Clinical Sciences, Linköping University, Linköping, Sweden
[24]Department of Psychiatry in Linköping, Linköping, Sweden
[25]Department of Psychiatry, University of Jena, Jena, Germany
[26] Institute of Translational Psychiatry, University of Muenster, Muenster, Germany
[27]Department of Psychology University of Halle, Halle, Germany

[28]*German Center for Mental Health (DZPG), Halle-Jena-Magdeburg, Magdeburg, Germany.*
[29]*Amsterdam Public Health Research Institute, Department of Mental Health, Amsterdam UMC, Amsterdam, The Netherlands*
[30]*GGZ Central Innova, Department of Research, Amersfoort, The Netherlands*
[31]*Department of Psychiatry and Psychotherapy, Central Institute of Mental Health (CIMH), Medical Faculty Mannheim, University of Heidelberg, Heidelberg, Germany*
[32]*Collaborative Antwerp Psychiatric Research Institute, University of Antwerp, Antwerpen, Belgium*
[33]*University Psychiatric Hospital Duffel, Duffel, Belgium*
[34]*Department of Psychiatry, Bellvitge University Hospital, Bellvitge Biomedical Research Institute- IDIBELL, Barcelona, Spain*
[35]*Department of Social Psychology and Quantitative Psychology, Universitat de Barcelona, Barcelona, Spain*
[36]*CIBERSAM, Carlos III Health Institute, Madrid, Spain*
[37]*Department of Psychiatry, Rijnstate Hospital Arnhem, Arnhem, The Netherlands*
[38]*Donders Institute for Brain, Cognition and Behavior, Department of Psychiatry, Radboud University Nijmegen, The Netherlands*
[39]*Department of Psychiatry, Bellvitge University Hospital, Bellvitge Biomedical Research Institute- IDIBELL, Barcelona, Spain*
[40]*Department of Clinical Sciences, Bellvitge Campus, Universitat de Barcelona, Barcelona, Spain*
[41]*Collaborative Antwerp Psychiatric Research Institute, University of Antwerp, Antwerpen, Belgium*
[42]*Psychiatric Hospital Bethanië, Zoersel, Belgium*
[43]*Amsterdam UMC location University of Amsterdam, Department of Psychiatry, Amsterdam, The Netherlands*
[44]*Amsterdam Neuroscience, Amsterdam, The Netherlands*
[45]*Service de Psychiatrie et Psychologie Médicale, Centre Expert Dépression Résistante, Foundation Fondamental, CHU, Toulouse, France*
[46]*Toulouse Neuroimaging Center, Université de Toulouse, INSERM, Toulouse, France*
[47]*Department of Psychiatry, Colombia School of Medicine, New York, USA*
[48]*Division of Psychology, Department of Psychiatry, UT Southwestern Medical Center, Dallas, Texas, USA*
[49]*Department of Clinical Medicine, University of Bergen, Bergen, Norway,*
[50]*Division of Psychiatry, Haukeland University Hospital, Bergen, Norway*
[51]*Department of Computer Science, University of Bergen, Bergen, Norway*
[52]*Department of Biological and Medical Psychology, University of Bergen, Bergen, Norway*
[53]*Department of Clinical Sciences Lund Psychiatry, Faculty of Medicine, Lund University, Lund, Sweden*
[54]*Department of Psychiatry, Skåne University Hospital, Lund, Sweden*
[55]*Department of Psychiatry, University of New Mexico, Albuquerque, New Mexico, USA*

Corresponding author
Olga Therese Ousdal, MD PhD
Department of Biomedicine, University of Bergen and Department of Radiology, Haukeland University Hospital, Jonas Lies vei 65, 5021 Bergen, Norway, Tel: +47 55 97 24 00 / Fax: +47 55975140, Email: olga.ousdal@uib.no/olga.therese.ousdal@helse-bergen.no

Supplementary Information

Supplementary Results

Volume changes, electric field and association with verbal fluency performance for the left hippocampus:

A within-group comparison of volumetric changes in patients revealed volumetric expansion of all left hippocampal subregions post-treatment (left hippocampal head: t=16.11, p_{fdr}=<0.001; left hippocampal body: t=12.10, p_{fdr}<0.001, left hippocampal tail: t=9.47, p_{fdr}<0.001; left total hippocampus: t=16.82, p_{fdr}<0.001). We next assessed the association between total or regional left hippocampal volumetric change following the electroconvulsive therapy (ECT) index series and change in verbal fluency (category and letter) while controlling for age, sex, site, number of ECTs and the respective baseline verbal fluency scores. While there was a trend toward a negative association between pre-post changes in category verbal fluency and changes in left hippocampal body volume (t=-1.51, p=0.10), no associations emerged between left subregional hippocampal volume changes and letter verbal fluency. In line with previous work from the GEMRIC[1, 2], there was a strong correlation between left total hippocampal volume change and the strength of the corresponding left hippocampal electric field in the total sample (r=0.27, t=5.55, p<0.001) and in patients receiving right unilateral stimulation only (r=0.25, t=4.50, p<0.001). However, the association did not remain when adjusting for age, sex, site, number of ECTs and electrode placement (i.e. right unilateral, bilateral or mixed) (all p> 0.05). Moreover, there were no significant associations between hippocampal head, body or tail volumetric changes and the strength of the corresponding regional EFs (all p> 0.05) after adjusting for the covariates listed above. Total and/or regional left hippocampal EFs were not associated with changes in category or letter verbal fluency following treatment (all p>0.05).

Associations between right hippocampal volumetric changes and changes in the Hopkins verbal learning test:

Due to the importance of the hippocampus in learning and memory, we additionally tested for associations between pre-post volumetric changes of the hippocampus and treatment-related changes in verbal learning. The specific test varied between sites, but we chose the Hopkins verbal learning test (HVLT) as we had most complete data for this test. All data on the HVLT were from one site. There were no significant changes in HVLT percent recall pre-post treatment (t=1.30, p=0.20 , N=42). Next, we assessed the association between whole or regional right hippocampal volume change and changes in HVLT percent recall while controlling for age, sex, number of ECTs and the baseline HVLT percent recall score. The analyses revealed no significant associations (all p>0.05).

Table S1: Letter and category verbal fluency versions separated by site

Site	Letter Fluency Version	Category Fluency Version
2		Animals
3		Animals
5		Animals
6	D	Animals
10	na / P,R,W	na / Animals
14	F,A,S	
16	F,A,S	Animals
17	P,M,R	Animals
20	na	na

Nine independent sites had included neurocognitive tests for category verbal fluency and/or letter verbal fluency before and after the ECT index series. Note that four sites only used tests of category verbal fluency and one site only included tests of letter verbal fluency. The version of the test used varied more for letter verbal fluence than category verbal fluency. For some sites the version number was not available (na).

Table S2: Medications

Medications	All Sites (N)
SSRI/SNRI	156
TCA	66
MAOI	2
Lithium	21
Antipsychotics	176
Medication free	96

SSRI: selective serotonin reuptake inhibitor, SNRI: serotonin and norepinephrine reuptake inhibitor, TCA: tricyclic antidepressant, MAOI: monoamine oxidase inhibitors

Table S3: Imaging acquisition details for the Global ECT MRI Research Collaboration (GEMRIC)

Site	Field strength	Vendor	TR (ms)	TE (ms)	Voxel-size (mm)
1	3	Siemens	2530	1.7-7.3	1.3 x 1 x 1
2	3	Philips	6.7	3.1	1.05 x 1.05 x 1.5
3	1.5	Philips	7.6	3.5	1.1 x 1.1 x 1.1
4	3	Siemens	1660	2.5	1.0 x 1.0 x 0.8
5	3	Siemens	2530	1.6	1 x 1 x 1
6	3	GE	7.8	3.0	1 x 1 x 1
7	3	Siemens	2530	1.7	1 x 1 x 1
8	3	Siemens	1900	1.7	1 x 1 x 1
9	3	Siemens	1570	2.75	1 x 1 x 1
10	3	Philips	8.1	3.7	1 x 1 x 1
11	3	Siemens	1380	2.6	1 x 1 x 1
12	3	Philips	7.4	3.4	0.5 x 0.5 x 0.5
13	3	Philips	13.3	6.1	0.6 x 0.5 x 0.5
14	3	Siemens	2500	4.4	1 x 1 x 1
15	3	GE	7.5	3	1 x 0.9 x 0.9
16	3	GE	6.5	2.8	1 x 1 x 1
17	3	Philips	8.1	3.7	0.9 x 0.9 x 1
18	3	GE	7.86	3.11	0.8 x 0.8 x 0.8
19	3	Siemens	2300	3	1 x 1 x 1
20	1.5	Siemens	2250	3.68	1 x 1 x 1
21	3	GE	6.9	2.9	0.9 x 0.9 x 1
22	3	Philips	9.6	4.6	1 x 1 x 1.2

Table S4: Volumetric changes of hippocampal longitudinal axis regions within patients

ROI	t	p	p_fdr
Right.Hippocampal.Head	24.5476	0.0000	0.0000
Right.Hippocampal.Body	15.8975	0.0000	0.0000
Right.Hippocampal.Tail	9.7996	0.0000	0.0000
Right.Whole.Hippocampus	24.5887	0.0000	0.0000

A paired-sample t-test was used to investigate within-subject volumetric changes pre-post treatment in patients. fdr: false discovery rate corrected.

Table S5: Volumetric changes of hippocampal longitudinal axis regions within healthy controls

ROI	t	p	p_fdr
Right.Hippocampal.Head	0.7595	0.4491	0.5987
Right.Hippocampal.Body	0.1518	0.8796	0.8796
Right.Hippocampal.Tail	0.9848	0.3268	0.5987
Right.Whole.Hippocampus	0.8218	0.4128	0.5987

A paired-sample t-test was used to investigate within-subject volumetric changes over the course of the study in controls. fdr: false discovery rate corrected.

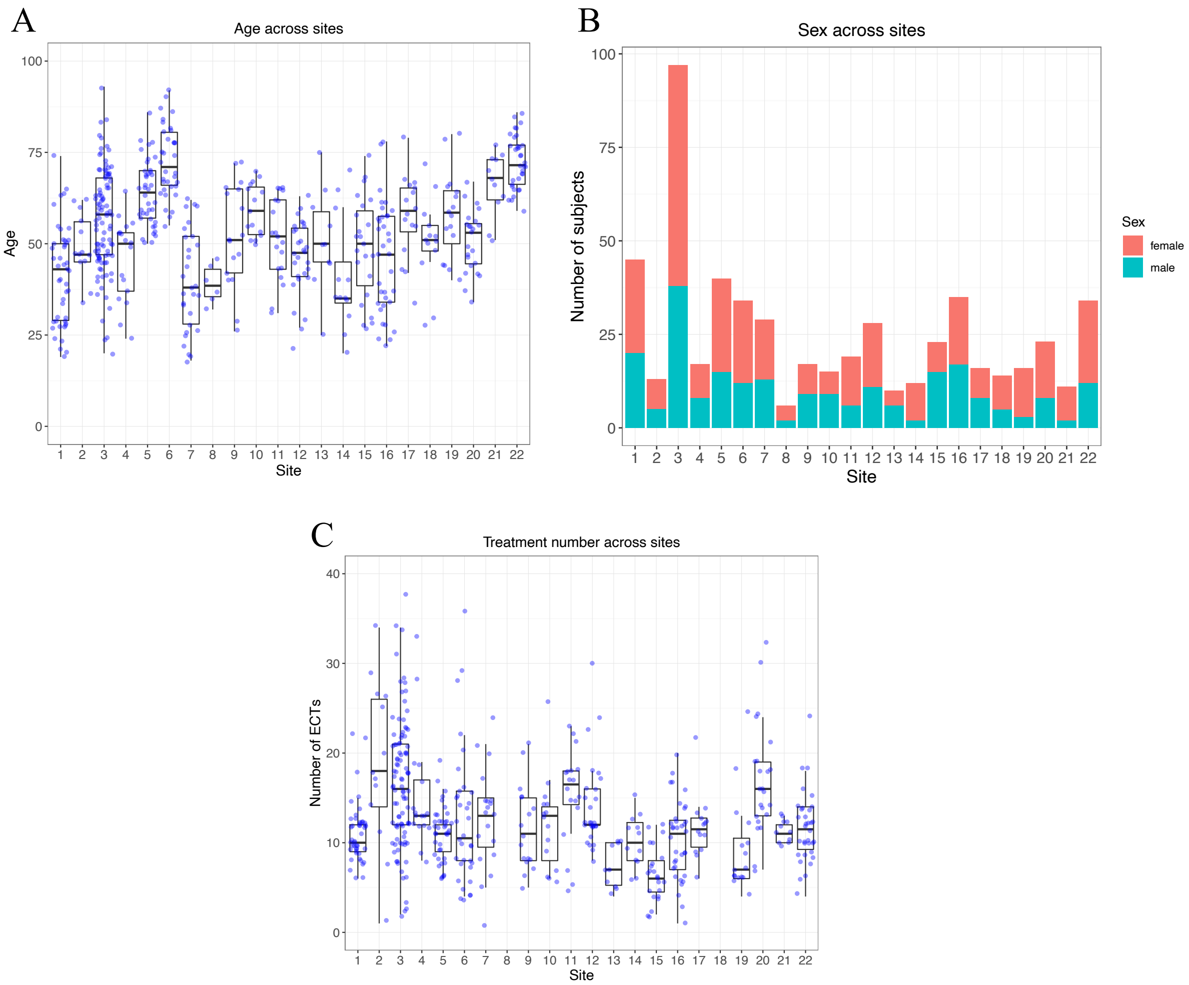

Figure S1. Patient clinical and demographic characteristics separated by site. A: Age of patients across sites. B: Sex distribution across sites C: Number of ECTs across sites

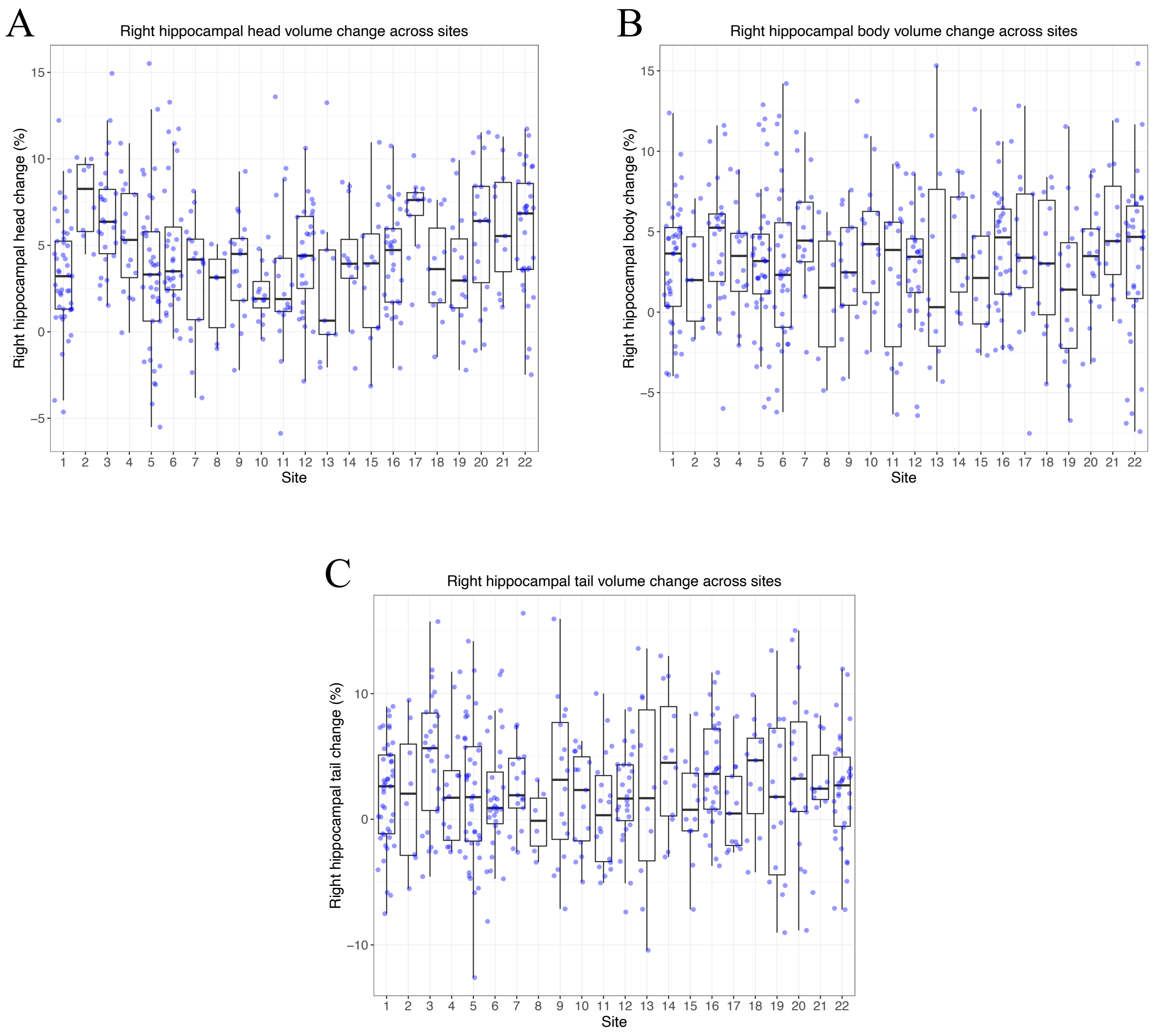

Figure S2. Hippocampal head, body and tail volumetric changes following electroconvulsive therapy (ECT) separated by site. A: Percentage volumetric change of the right hippocampal head ($\Delta Vol_{r\text{-hippocampal head}}/Pre\text{-}ECT\text{-}Vol_{r\text{-hippocampal head}}$) following ECT across sites. B: Percentage volumetric change of the right hippocampal body ($\Delta Vol_{r\text{-hippocampal body}}/Pre\text{-}ECT\text{-}Vol_{r\text{-hippocampal body}}$) following ECT across sites. C: Percentage volumetric change of the right hippocampal tail ($\Delta Vol_{r\text{-hippocampal tail}}/Pre\text{-}ECT\text{-}Vol_{r\text{-hippocampal tail}}$) following ECT across sites.

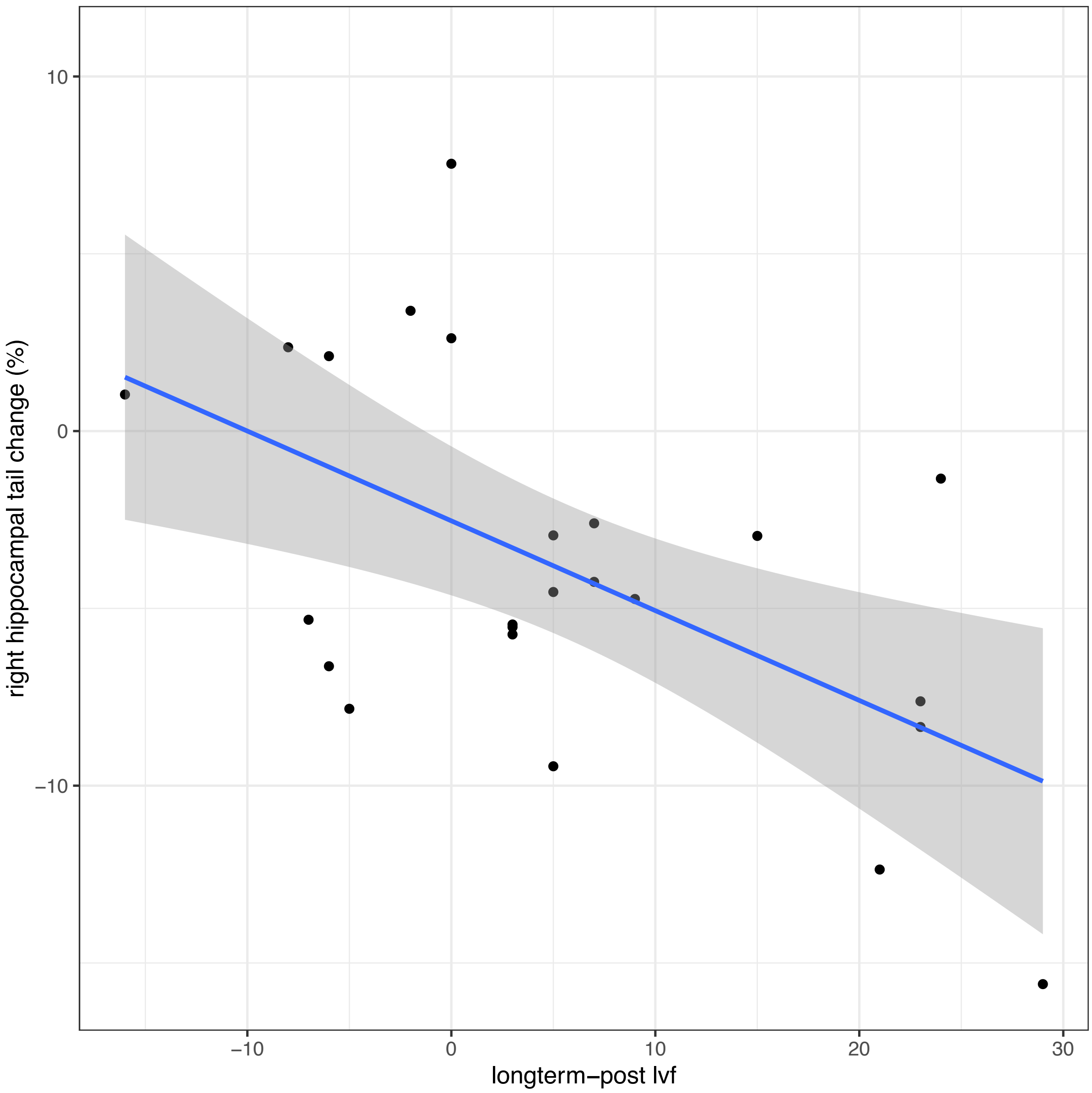

Figure S3. Long-term volumetric normalization of the hippocampal tail and long-term cognitive performance. Scatter plot of the association between percentage long-term volumetric reduction of the right hippocampal tail ($(Vol_{6\ months\ follow\ up}$ - $Vol_{post\text{-}treatment})/Vol_{post\text{-}treatment})$ and change in letter verbal fluency performance (lvf: performance at 6 months follow up – performance post-treatment) ($t=-2.8$, $p_{fdr}<0.05$). The regression line (with 95% confidence interval shown as shaded area) represent the relationship between the dependent and the independent variable calculated without covariates.

References

1. Argyelan M, Oltedal L, Deng ZD, Wade B, Bikson M, Joanlanne A et al. Electric field causes volumetric changes in the human brain. Elife 2019; 8: e49115.

2. Argyelan M, Deng ZD, Ousdal OT, Oltedal L, Angulo B, Baradits M et al. Electroconvulsive therapy-induced volumetric brain changes converge on a common causal circuit in depression. Mol Psychiatry 2024; 29(2): 229-237.
