# Supplementary material for: Effects of electroconvulsive therapy on hippocampal longitudinal axis and its association with cognitive side effects
Source: Commun Med (Lond). 2025 Oct 1;5:409. doi: 10.1038/s43856-025-01120-1 (PMC12489027; doi:10.1038/s43856-025-01120-1)
Supplement: Supplementary file 3 — Reporting Summary [file 43856_2025_1120_MOESM3_ESM.pdf]

nature portfolio

Corresponding author(s): COMMSMED-24-1126-T
Last updated by author(s): Oct 29, 2024

Reporting Summary

Nature Portfolio wishes to improve the reproducibility of the work that we publish. This form provides structure for consistency and transparency in reporting. For further information on Nature Portfolio policies, see our Editorial Policies and the Editorial Policy Checklist.

Statistics

For all statistical analyses, confirm that the following items are present in the figure legend, table legend, main text, or Methods section.

n/a	Confirmed	
☐	☒	The exact sample size (*n*) for each experimental group/condition, given as a discrete number and unit of measurement
☐	☒	A statement on whether measurements were taken from distinct samples or whether the same sample was measured repeatedly
☐	☒	The statistical test(s) used AND whether they are one- or two-sided<br>*Only common tests should be described solely by name; describe more complex techniques in the Methods section.*
☐	☒	A description of all covariates tested
☐	☒	A description of any assumptions or corrections, such as tests of normality and adjustment for multiple comparisons
☐	☒	A full description of the statistical parameters including central tendency (e.g. means) or other basic estimates (e.g. regression coefficient) AND variation (e.g. standard deviation) or associated estimates of uncertainty (e.g. confidence intervals)
☐	☒	For null hypothesis testing, the test statistic (e.g. *F*, *t*, *r*) with confidence intervals, effect sizes, degrees of freedom and *P* value noted<br>*Give P values as exact values whenever suitable.*
☒	☐	For Bayesian analysis, information on the choice of priors and Markov chain Monte Carlo settings
☒	☐	For hierarchical and complex designs, identification of the appropriate level for tests and full reporting of outcomes
☐	☒	Estimates of effect sizes (e.g. Cohen's *d*, Pearson's *r*), indicating how they were calculated

Our web collection on statistics for biologists contains articles on many of the points above.

Software and code

Policy information about availability of computer code

Data collection	GEMRIC dataset (datarelease 3.2, DOI 10.17605/OSF.IO/YP2G4)
Data analysis	R studo

For manuscripts utilizing custom algorithms or software that are central to the research but not yet described in published literature, software must be made available to editors and reviewers. We strongly encourage code deposition in a community repository (e.g. GitHub). See the Nature Portfolio guidelines for submitting code & software for further information.

Data

Policy information about availability of data

All manuscripts must include a data availability statement. This statement should provide the following information, where applicable:

- Accession codes, unique identifiers, or web links for publicly available datasets
- A description of any restrictions on data availability
- For clinical datasets or third party data, please ensure that the statement adheres to our policy

All code used in this study will be made available upon reasonable request. The datasets generated and analysed during the current study are available to members of the GEMRIC consortium, in accordance with the consortium's data sharing policies and subject to approval by the relevant ethics committee and the institution's data protection officer.

Human research participants

Policy information about studies involving human research participants and Sex and Gender in Research.

Reporting on sex and gender	We report the biological sex, no information about gender identity have been collected.
Population characteristics	Covariates included: Age, sex, site, number of ECTs, Electrode placement, baseline performance on relevant test
Recruitment	Mutli-site study. Most patients were recruited as part of specialist mental health care.
Ethics oversight	Regional Ethics Committee South-East Norway (#2018/769).

Note that full information on the approval of the study protocol must also be provided in the manuscript.

Field-specific reporting

Please select the one below that is the best fit for your research. If you are not sure, read the appropriate sections before making your selection.

☒ Life sciences ☐ Behavioural & social sciences ☐ Ecological, evolutionary & environmental sciences

For a reference copy of the document with all sections, see nature.com/documents/nr-reporting-summary-flat.pdf

Life sciences study design

All studies must disclose on these points even when the disclosure is negative.

Sample size	Multisite mega-analyses including data from 22 sites (435 patients with a major depressive episode and 119 healthy controls)
Data exclusions	No exclusion
Replication	Used related cognitive tests to test for generalizability of findings
Randomization	Not relevant
Blinding	Not relevant

Reporting for specific materials, systems and methods

We require information from authors about some types of materials, experimental systems and methods used in many studies. Here, indicate whether each material, system or method listed is relevant to your study. If you are not sure if a list item applies to your research, read the appropriate section before selecting a response.

Materials & experimental systems

n/a	Involved in the study	
☒	☐	Antibodies
☒	☐	Eukaryotic cell lines
☒	☐	Palaeontology and archaeology
☒	☐	Animals and other organisms
☐	☒	Clinical data
☒	☐	Dual use research of concern

Methods

n/a	Involved in the study	
☒	☐	ChIP-seq
☒	☐	Flow cytometry
☐	☒	MRI-based neuroimaging

Clinical data

Policy information about clinical studies

All manuscripts should comply with the ICMJE guidelines for publication of clinical research and a completed CONSORT checklist must be included with all submissions.

Clinical trial registration	Not a clinical trial
Study protocol	Not a clinical trial

Data collection	Not a clinical trial
Outcomes	Not a clinical trial

Magnetic resonance imaging

Experimental design

Design type	Structural MRI
Design specifications	Not relevant
Behavioral performance measures	Not relevant

Acquisition

Imaging type(s)	Structural, T1 weighted
Field strength	1.5 or 3T
Sequence & imaging parameters	Please see Supplementary Table 3
Area of acquisition	Whole brain scan

Diffusion MRI ☐ Used ☒ Not used

Preprocessing

Preprocessing software	FreeSurfer Version 7.1.1
Normalization	Standard recon-all pipeline
Normalization template	Not relevant
Noise and artifact removal	Not relevant
Volume censoring	Not relevant

Statistical modeling & inference

Model type and settings	Not relevant
Effect(s) tested	No fMRI study

Specify type of analysis: ☐ Whole brain ☒ ROI-based ☐ Both

Anatomical location(s)	Hippocampus, including the head, body and tail subdivisions
Statistic type for inference (See Eklund et al. 2016)	Region of interest
Correction	FDR

Models & analysis

n/a	Involved in the study
☒	☐ Functional and/or effective connectivity
☒	☐ Graph analysis
☒	☐ Multivariate modeling or predictive analysis
